# Transurethral resection of ureteral tumors at the intramural segment: A pilot study

**DOI:** 10.14440/bladder.2025.0009

**Published:** 2025-09-02

**Authors:** Yi Wang, Huiqing Wang, Chen Ye, Zhensheng Zhang, Shuxiong Zeng, Chuanliang Xu

**Affiliations:** 1 Department of Urology, Changhai Hospital, Naval Medical University, Shanghai 200433, China; 2 Department of Urology, Shanghai General Hospital, Shanghai Jiao Tong University School of Medicine, Shanghai 201613, China

**Keywords:** Intramural ureter, Kidney-preserving surgery, Transurethral resection of urothelial tumors, Upper urinary tract urothelial carcinoma

## Abstract

**Background:**

Ureteral tumors are rare and present unique diagnostic and therapeutic challenges.

**Objective:**

This study aimed to evaluate the efficacy and safety of transurethral resection (TUR) in treating ureteral tumors located in the intramural segment of the bladder wall.

**Methods:**

This retrospective study analyzed the clinical data of 24 patients who underwent TUR for intramural ureteral tumors at Changhai Hospital, Shanghai, China, from May 2020 to September 2023. All patients were treated using an “outer wedge, inner rotation” technique, with ureteral stent placed during the procedure.

**Results:**

All surgeries were successfully completed, with a median operative time of 45 min (range: 25–75 min), median intraoperative blood loss of 5 mL (range: 1–30 mL), median post-operative hospitalization of 4 days (range: 3–10 days), mean ureteral stent placement duration of 34.1 ± 10.1 days, and a mean tumor resection diameter of 1.9 ± 0.7 cm. There were no intraoperative complications, while post-operative hydronephrosis occurred in 12.5% of patients. Pathological examination revealed that all tumors were urothelial carcinoma, with five cases of Ta stage, one case of pT1 low-grade, 14 cases of pT1 high-grade, and four cases of pT2a high-grade. The median follow-up period lasted for 28 months (range: 11–38 months), with a tumor recurrence rate of 20.8%.

**Conclusion:**

TUR of ureteral tumors in the intramural segment is a minimally invasive procedure associated with low blood loss, reduced post-operative complications, and efficacious tumor control while preserving renal function. However, the possibility of local tumor recurrence remains, necessitating close post-operative surveillance.

## 1. Introduction

Upper urinary tract urothelial carcinoma (UTUC), which includes renal pelvis and ureteral carcinomas, represents a relatively rare urological malignancy, accounting for only 5–10% of all urothelial cancers.^1^,^2^ Despite its low incidence, UTUC is known for its aggressive nature and poor prognosis.^2^ Ureteral carcinoma has historically been less common in clinical practice, with its incidence being approximately half that of renal pelvis carcinoma. However, recent trends indicated a noticeable increase in the incidence of ureteral carcinoma in China.^3^,^4^ Radical nephroureterectomy remains the gold standard for surgical treatment, involving the removal of the kidney, the entire ureter, and a part of the bladder wall. Traditional treatments are effective, but are often associated with significant morbidity and a high rate of complications. Recent studies reported that approximately 30% of patients who underwent nephroureterectomy developed significant complications, such as renal failure and hydronephrosis.^5^,^6^

For patients with low-risk ureteral carcinoma and high-risk distal ureteral carcinoma, recent studies suggested that kidney-preserving ureteral segmental resection or endoscopic tumor resection can preserve renal function without compromising the patient’s prognosis.^7^ This approach is particularly beneficial for patients with tumors situated at the intramural segment of the ureter, where localized surgery may offer greater advantages.^8^,^9^

Presented in this study were the clinical outcomes of 24 patients who underwent transurethral resection (TUR) of ureteral tumors at the intramural segment of the bladder wall (stages Ta–T2) in the Department of Urology at Changhai Hospital, Shanghai, China, between May 2020 and September 2023. The novel “outer wedge, inner rotation” surgical strategy was described, in which a wedge-shaped incision is made at the ureteral orifice, followed by rotational resection of the tumor within the intramural segment of the ureter. This study aimed to explore the clinical efficacy and safety of this new surgical approach.

## 2. Materials and methods

### 2.1. Clinical data

Included were 24 patients who underwent TUR of urothelial carcinoma in the intramural segment of the ureter at Changhai Hospital, Shanghai, from May 2020 to September 2023 and their data were retrospectively analyzed. The inclusion criteria were as follows: (i) ability to tolerate general anesthesia and voluntarily opted for TUR of intramural ureteral tumors; (ii) presence of a mass in the intramural segment of the ureter confirmed by pre-operative cystoscopy, with no signs of invasive growth or regional lymph node metastasis on pre-operative imaging; (iii) provision of signed informed consent for surgery; and (iv) completion of a post-operative follow-up period of more than 6 months.

The exclusion criteria included: (i) presence of *in situ* carcinoma within the bladder, with tumors invading the muscle layer, lymph node, or having distant metastases, or other concomitant malignancies; (ii) priorly having received neoadjuvant chemoradiotherapy; (iii) suffering from severe cardiac or pulmonary dysfunction, serious infection, coagulopathy, or other severe chronic underlying diseases; (iv) conditions affecting surgical treatment, such as post-kidney transplantation or dialysis dependence; and (v) incomplete clinical data.

This surgical technique is indicated for patients with ureteral tumors located in the intramural segment of the bladder wall, where complete resection is feasible without compromising renal function. Contraindications include tumors extending beyond the bladder wall or patients with significant comorbidities that may complicate the procedure. All 24 patients had tumors situated in the intramural segment of the ureter; with tumors on the left side in 11 cases and on the right side in 13 cases. Computed tomography (CT) urography revealed that the diameter of the all tumors measured <2 cm, with no evidence of invasive growth or regional lymph node metastasis. All patients underwent TUR of ureteral tumors in the intramural segment of the bladder wall. The general characteristics of the patients are shown in Table 1.

**Table 1:** Patient characteristics

Variable	Number (*n*=24)
Gender, *n* (%)	
Male	19 (79.2)
Female	5 (20.8)
Age (year)	68.5±10.4
BMI (kg/m^2^	23.9±3.5
Hematuria *n* (%)	
Yes	18 (75.0)
No	6 (25.0)
Pre-operative hydronephrosis *n* (%)	2 (8.3)

Abbreviation: BMI: Body mass index.

### 2.2. Surgical procedure

**Figure 1:**
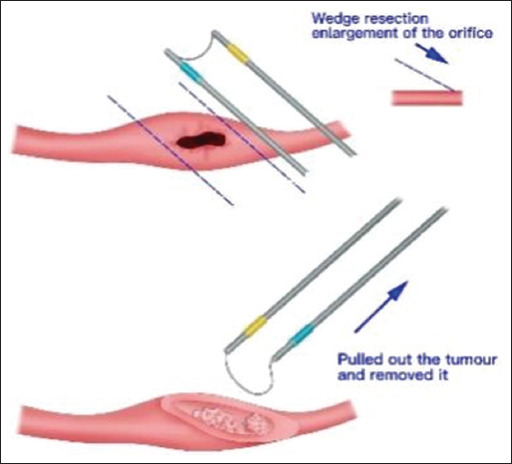
Schematic of the operation

**Figure 2:**
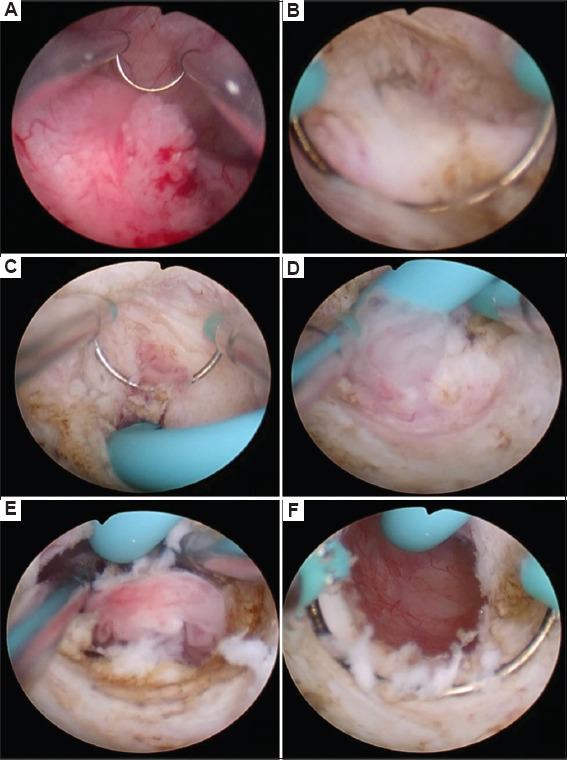
Intraoperative screenshots. (A) Tumor at the right ureteral orifice, bulging into the bladder. (B) Wedge excision of the ureteral orifice to enlarge the ureteral orifice. (C) Placement of a ureteral stent. (D) Using the ureteral stent as an axis, the abnormal mucosa of the ureter is excised. (E) Proceed to deepen the mucosal excision until normal mucosa is visible. (F) Outcomes following transurethral resection of intramural ureteral tumors

After the induction of general anesthesia, the patient was positioned in the lithotomy position, and the surgical area was disinfected and draped. A 24F or 26F resectoscope was inserted transurethrally to inspect the entire urethra and bladder. Using an electrocautery loop, an incision was made 1.5 cm above the affected ureteral orifice along the course of the ureter, extending to the muscle layer of the bladder wall in a wedge-shaped fashion. The widened ureteral orifice resembled an oblique cut on a bamboo tube. The superficial portion of the ureteral tumor extending into the bladder was resected with the electrocautery loop (Figure 1). A ureteral stent was placed under direct vision to prevent ureteral detachment due to the resection. Using the stent as a guide, the tumor in the intramural ureter was resected in a rotational manner, similar to TUR of the prostate, removing the deeper layers of the tumor down to the muscle. Surgical margins and the tumor base were biopsied separately for pathological examination. Hemostasis was achieved using electrocautery. Antibiotics were administered to prevent infection (Figure 2).

Within 24 h post-operation, patients received a single instillation of intravesical chemotherapy with agents such as pirarubicin or gemcitabine. The catheter was removed on the 5^th^ post-operative day unless intraoperative perforation occurred. In the case of intraoperative perforation, the catheter was left in place for 7–10 days, up to a maximum of 14 days. The ureteral stent was removed after 1 month, though in the case of post-operative hydronephrosis, the stent was retained for up to 2 months.

### 2.3. Tumor grading, staging, and patient follow-up

Tumors were graded according to the 2004/2016 World Health Organization classification system,^10^ and staging was based on the 2017 Tumor-Node-Metastasis classification for UTUC.^11^ Post-operative follow-up was conducted according to a standardized protocol, with patients being monitored every 3 months for the first year and then biannually thereafter. Follow-up assessments included detection of any recurrence or complications by cystoscopy, computed tomography (CT) imaging, and renal function tests.

### 2.4. Observational indicators and statistical methods

We recorded the intraoperative procedures, complications, and oncological outcomes of 24 patients who underwent TUR of ureteral tumors in the intramural segment. Observational parameters involved pre-operative hydronephrosis, operative time, intraoperative blood loss, intraoperative complications, post-operative hydronephrosis, pathological results, and tumor recurrence rates. Data were analyzed using the Statistical Package for the Social Sciences 22.0 software package (IBM, United States). Continuous variables were presented as mean ± standard deviation, and comparisons between two groups were made using the *t*-test. Categorical variables were analyzed using the chi-square test. Statistical significance was defined as *p*<0.05.

## 3. Results

All 24 surgeries were successfully completed, with no conversions to open surgery. The median operative time lasted for 45 min (range: 25–75 min), and the median intraoperative blood loss was 5 mL (range: 1–30 mL). The average tumor resection diameter was 1.9 ± 0.7 cm. No intraoperative complications, such as bladder perforation or ureteral detachment, were observed. The median hospital stay was 4 days (range: 3–10 days). The median follow-up duration was 28 months (range: 11–38 months). In addition, the average ureteral stent placement duration was 34.1 ± 10.1 days. Postoperatively, three patients developed new-onset upper urinary tract hydronephrosis. No other significant post-operative complications, such as marked hydronephrosis, vesicoureteral reflux, or acute pyelonephritis, were observed in the remaining patients during follow-up.

Post-operative pathology confirmed urothelial carcinoma in all 24 patients, with five cases classified as pTa, one as pT1 low-grade, 14 as pT1 high-grade, and four as pT2a high-grade. Five patients (20.8%) experienced tumor recurrence, with high-grade T1 tumors exhibiting the highest recurrence (35.7%). Details of tumor recurrence stratified by pT stages and grades are shown in Table 2. Among the patients, one patient developed a recurrence at the distal end of the ipsilateral ureter and subsequently underwent radical nephroureterectomy, three patients experienced bladder tumor recurrence – with one receiving TUR of bladder tumor (TURBT) and two undergoing radical cystectomy – and one patient developed distant metastasis to the vertebrae. There were no tumor-related deaths during the follow-up period (Table 3).

**Table 2:** Recurrence rates by pathological stages and grades

pT stage	Grade	Recurrence rate (%)
Ta	Low	0/5 (0)
T1	Low	0/1 (0)
T1	High	5/14 (35.7)
T2a	High	0/4 (0)

**Table 3:** Post-operative characteristics

Variable	Number (*n*=24)
^a^Operation time (minutes)	45 (25–75)
T stage, *n* (%)	
Ta	5 (20.8)
T1	15 (62.5)
T2	4 (16.7)
Grade, *n* (%)	
L	6 (25.0)
H	18 (75.0)
^a^Hospitalization time (day)	4 (3–10)
^b^Ureteral stent placement duration (days)	34.1±10.1
Post-operative hydronephrosis, *n* (%)	3 (12.5)
Recurrence, *n* (%)	5 (20.8)

Note: Data are presented as

a median (range) and

b mean±standard deviation, unless otherwise specified.

## 4. Discussion

Primary ureteral carcinoma is relatively rare. However, with a growing aging population and advancements in imaging, cytology, and endoscopic techniques, the cases of UTUC have been on rise significantly in recent years.^3^ Radical nephroureterectomy has long been the standard surgical treatment for ureteral carcinoma.^12^,^13^ However, this procedure involves extensive resection, significant surgical trauma, and a higher incidence of post-operative complications, such as chronic renal dysfunction and cardiovascular events. Approximately 25% of patients may lose eligibility for chemotherapy due to deteriorating renal function, negatively impacting overall survival.^14^ Kidney-preserving surgery (KPS) offers an alternative for patients with poor baseline renal function, allowing for more treatment options. The main kidney-preserving procedures include ureteral segmental resection with end-to-end anastomosis, distal ureterectomy with ureteral reimplantation, holmium or thulium laser excision, and percutaneous nephroscopic tumor resection.

Several retrospective studies have demonstrated that, there existed no significant difference in 5-year cancer-specific survival between the low-risk patients receiving KPS and their counterparts undergoing radical surgery.^8^,^15^ The latest European Association of Urology (EAU) guidelines classify patients with single, small, low-grade, and non-invasive lesions as having low-risk UTUC, recommending KPS regardless of contralateral renal function status. In high-risk patients, such as those with renal insufficiency or a solitary functional kidney, KPS may also be considered following careful evaluation.^4^ In addition, studies have shown that endoscopic KPS is less invasive than ureteral segmental resection while achieving similar efficacy.^7^

The “TUR of ureteral tumors in the intramural segment” described in this study falls under the category of kidney-preserving endoscopic procedures. It is a novel surgical approach targeting ureteral tumors located in the bladder’s intramural segment, a unique subtype of ureteral cancer. These tumors tend to present with symptoms early such as hydronephrosis and hematuria and are typically diagnosed at an early stage when they are still within the range of TUR. Consistent with the EAU 2023 Guidelines and the American Urological Association recommendations, kidney-sparing surgery (KSS) for high-risk UTUC carries a higher risk of disease progression compared to low-risk UTUC, potentially impacting long-term survival.^4^,^16^ Therefore, the TUR technique described herein, as a form of KSS, should be considered for high-risk UTUC (*e*.*g*., pT1 high-grade and pT2a) only on a case-by-case basis, specifically in patients with imperative indications, such as a solitary kidney, bilateral UTUC, severe chronic renal disease, or inability to tolerate radical nephroureterectomy.

In recent years, endoscopic surgeries using ureteroscopes and resectoscopes have been incrementally applied for the treatment of ureteral carcinoma. In our experience, the intramural ureter is tough and elastic, rendering it challenging to dilate the ureteral lumen with the flexible laser fibers. In contrast, the electrocautery loop used in this study, being supported and protected by a ureteral stent, allows for blunt dilation and deeper access to the tumor within the intramural ureter. Given the narrow lumen of the intramural ureter, we recommend using a 24F or 26F resectoscope for better maneuverability during the procedure. Mano *et al*.^17^ reported that 35% of patients undergoing TURBT had tumors covering the ureteral orifice or located too close to the orifice, requiring resection of the ureteral orifice. Therefore, for tumors in the intramural segment of the ureter, especially those protruding into the bladder and causing ureteral orifice stricture and upper urinary tract dilation, TUR is a highly feasible treatment option.

The TUR approach offers several advantages over traditional methods:

(i) A low risk of post-operative ureteral stricture. Although electrocautery may cause scarring and ureteral stricture at the orifice, the wedge-shaped incision extending to the muscle layer of the intramural ureter helps to enlarge the ureteral opening, reducing the likelihood of stricture. In this study, three patients developed mild post-operative upper urinary tract hydronephrosis, but there was no deterioration of symptoms during follow-up.
(ii) Simultaneous management of bladder tumors. Ureteral tumors in the intramural segment are often located in the vicinity of the ureteral orifice, usually involving bladder. Studies have shown that ureteral cancer tends to recur in regions distal to the original tumor site, with bladder tumor recurrence being common in intramural ureteral tumors.^18^,^19^ This technique allows for the resection and sampling of any suspicious lesions in the bladder during the procedure, lowering the likelihood of missed bladder lesions.
(iii) Low incidences of post-operative complications. The Waldeyer’s sheath surrounding the intramural ureter, along with the bladder’s detrusor muscle, provides a dual support, preventing reflux of urine into the ureter during voiding. Among the 24 patients in this study, no significant intraoperative complications took place, such as massive bleeding, bladder perforation, or ureteral detachment, and no vesicoureteral reflux was detected during follow-up.

Tumor recurrence remains a key concern in the clinical management of ureteral carcinoma. The recurrence rate after ureteroscopic holmium laser treatment of UTUC is reportedly 31–65%.^20^ This high recurrence rate may be ascribed to the increased intraluminal pressure during ureteroscopy, which increases the risk of tumor cell dissemination. The peak periods for bladder tumor recurrence after ureteral cancer surgery occur at 4–6 months and 17–19 months postoperatively, after which recurrence rates drop gradually.^18^ In this study, with a median follow-up of 28 months (range: 11–38 months), 5 patients (20.8%) experienced tumor recurrence, which is lower than the rates reported for laser treatment.

A retrospective study by Rodriguez Faba *et al.*,^21^ involving patients who underwent TUR of intramural ureteral tumors, reported that pT1 staging and tumor size ≥1.5 cm were independent predictors of post-operative ureteral stricture, and patients with pT1 stage or carcinoma *in situ* were at a higher risk of upper urinary tract tumor recurrence. Therefore, in patients undergoing KPS for ureteral tumors in the intramural segment, careful attention should be paid to post-operative pathology, intravesical instillation of chemotherapeutics, and regular follow-up with CT and cystoscopy to monitor for recurrence. In this study, in the five patients who experienced tumor recurrence, all were high-grade pT1 on post-operative pathology. Only one patient developed distant metastasis to the thoracic spine, while the remaining patients suffered from local recurrences in the bladder or the distal ureter. Notably, the patient with thoracic spine metastasis maintained good renal function during systemic chemotherapy.

In addition, Li *et al*.^22^ reported that patients with high-grade urothelial carcinoma of the ureter who underwent segmental ureterectomy followed by post-operative radiotherapy had a significantly improved recurrence-free survival (67.6% *vs*. 39.5%, hazard ratio = 2.431, 95% confidence interval [CI] = 1.210–4.883, *p*=0.039) compared to those who did not receive radiotherapy, with no significant difference found in overall survival compared to radical nephrectomy (67.6% *vs*. 64.4%, hazard ratio = 1.113, 95% CI 0.457–2.712, *p*=0.821). Future treatments for high-grade urothelial carcinoma may benefit from the application of post-operative radiotherapy to minimize tumor recurrence and expand the applicability of KPS.

## 5. Conclusion

This study presents a novel surgical technique for treating ureteral tumors in the intramural segment. The wedge-shaped incision of the intramural ureter, combined with rotational resection, ensures precise tumor removal with minimal intraoperative and post-operative complications. Compared to traditional ureteral segmental resection, this approach offers a less invasive option that preserves patient quality of life and is more suitable for elderly patients or those with multiple comorbidities who cannot tolerate kidney removal surgery. The “outer wedge, inner rotation” technique is also easy to learn and perform. Despite the promising results, several limitations of this study must be acknowledged. The retrospective design may introduce selection bias, and the small sample size limits the generalizability of the findings. In addition, the relatively short follow-up period restricts the ability to assess long-term outcomes such as recurrence rates and renal function preservation.

## Data Availability

The data used in this study can be obtained by contacting the corresponding authors. Upon receiving reasonable requests from readers, the authors will provide the corresponding data in accordance with relevant regulations and procedures to ensure the standardized use and sharing of the data.
